# Data-Driven Dietary Patterns and Diet Quality Scores: Reproducibility and Consistency in Sex and Age Subgroups of Poles Aged 15–65 Years

**DOI:** 10.3390/nu12123598

**Published:** 2020-11-24

**Authors:** Joanna Kowalkowska, Lidia Wadolowska, Jolanta Czarnocinska, Grzegorz Galinski, Anna Dlugosz, Dorota Loboda, Magdalena Czlapka-Matyasik

**Affiliations:** 1 Department of Human Nutrition, University of Warmia and Mazury in Olsztyn, Słoneczna 45F, 10-718 Olsztyn, Poland; lidia.wadolowska@uwm.edu.pl; 2 Department of Human Nutrition and Dietetics, Poznan University of Life Sciences, Wojska Polskiego 28, 60-637 Poznan, Poland; jolanta.czarnocinska@up.poznan.pl (J.C.); grzegorz.galinski@up.poznan.pl (G.G.); magdalena.matyasik@up.poznan.pl (M.C.-M.); 3 Faculty of Chemical Technology and Engineering, University of Technology and Life Sciences in Bydgoszcz, Seminaryjna 3, 85-326 Bydgoszcz, Poland; anna.dlugosz@utp.edu.pl; 4 Institute of Health, University of Economy in Bydgoszcz, Garbary 2, 85-229 Bydgoszcz, Poland; dorota.loboda@byd.pl

**Keywords:** FFQ, dietary assessment, diet quality, dietary pattern, PCA, reproducibility, reliability, adolescents, adults

## Abstract

This study aimed to assess: (i) the test–retest reproducibility of identification of data-driven dietary patterns (DPs) derived using a Principal Component Analysis (PCA) and hypothesis-driven DPs (diet quality scores); (ii) the consistency of data-driven DPs with diet quality scores in sex and age subgroups of Poles aged 15–65 years. The study involved 504 subjects (55.6% of females). Data on food consumption frequency (33 food items) were collected twice with a two-week interval using the Dietary Habits and Nutrition Beliefs Questionnaire (KomPAN^®^) in a self-administered version (test and retest). Two major data-driven DPs (‘Prudent’ and ‘Western’) were identified in the total sample, sex groups and four age groups separately from test and retest data. Two diet quality scores were analysed: Pro-Healthy-Diet-Index-10 (pHDI-10) and Non-Healthy-Diet-Index-14 (nHDI-14). Tucker’s congruence coefficient indicated fair-to-good similarity of data-driven DPs between test and retest for all study subgroups, except for males. Across study subgroups, the intraclass correlation coefficient (ICC) between the test and retest ranged from 0.56 to 0.86 for ‘Prudent’ DP and 0.57 to 0.82 for ‘Western’ DP, with the lowest values in males. The ICC (test vs. retest) ranged from 0.84 to 0.88 for pHDI-10 and 0.75 to 0.88 for nHDI-14. Comparing the data-driven DPs and diet quality scores, the Spearman’s correlations ranged from 0.63 to 0.93 between ‘Prudent’ DP and pHDI-10, and from 0.60 to 0.81 between ‘Western’ DP and nHDI-14. The test–retest reproducibility of data-driven DPs and diet quality scores and their consistency were acceptable in most of the study subgroups, with a tendency to be higher for pro-health than unhealthy DPs. Data-driven DPs were more reproducible in females than males. The reproducibility of diet quality scores tended to be better in males than females and was the highest in 25–44-year-olds. The KomPAN^®^ questionnaire can be recommended to use data-driven DPs and diet quality scores to describe the habitual diet in people aged 15–65 years.

## 1. Introduction

Dietary patterns (DPs) enable a comprehensive assessment of the habitual diet and are widely used in epidemiological studies [1,2,3,4,5]. Identification of DPs emphasizes the importance of holistic evaluation of the diet as different foods are consumed in complex combinations and their synergistic effect on health should be considered in assessing the diet-disease relationship [6,7,8,9]. DPs can be derived using an a priori (hypothesis-driven) or an a posteriori (data-driven) approach [7,8,9,10,11,12,13,14,15]. Hypothesis-driven DPs (diet quality scores) are developed based on dietary recommendations and reflect adherence to healthy (or unhealthy) eating habits [8,14,16]. The number of items included in the diet quality score is one of the key decisions of the researcher [8,10,13,14]. Data-driven DPs are identified using statistical methods, for example: cluster analysis (CA), principal component analysis (PCA), explanatory factor analysis (EFA) [5,7,9,11,13,15,17]. Data-driven DPs can combine healthy and less healthy foods (or dietary characteristics) [2,4,16,18]. Although data-driven DPs may not be as polarised as hypothesis-driven DPs, they can more accurately reflect real eating habits as combinations of food consumed in different groups of the population [10,16].

An accurate dietary assessment is crucial in evaluating the relation between diet and health. Food frequency questionnaires (FFQs) assess the habitual diet over a long period of time (e.g., a year) and are commonly used tools in nutrition research due to their simplicity and cost-effectiveness [19,20,21]. Each newly created or adapted FFQ should be tested for reliability, including test–retest reproducibility [13,19]. Reproducibility is influenced by random errors in dietary assessment and is evaluated by comparing the results from both administrations of the questionnaire (test and retest) [22]. Besides assessing the test–retest reproducibility of food items included in the questionnaire, it is important to evaluate the reproducibility of DPs derived based on the FFQ data. Numerous factors, such as sex, age, socio-economic status and region of residence influence food choices and are associated with DPs [1,2,3,12]. DPs identified in different groups of people can vary and have different levels of reproducibility [17].

There are a limited number of studies in the world on the test–retest reproducibility of diet quality scores [23,24,25,26,27] or data-driven DPs [6,28,29,30,31,32] and few have been conducted in Poland [33,34]. One study showed findings on the test–retest reproducibility of diet quality scores in healthy and unhealthy subjects as well as for two modes of administration of the questionnaire (interview-administered and self-administered) [33]. Another study was related to the reproducibility of data-driven DPs identified in females aged 13–21 years [34]. To the best of our knowledge, there have been no studies on the test–retest reproducibility of DPs identified separately in sex or age subgroups of Poles. Since the Dietary Habits and Nutrition Beliefs Questionnaire (KomPAN^®^) was developed for people in a wide age range (15–65 years), it is crucial to assess the test–retest reproducibility and consistency of DPs derived using two approaches in different subgroups of people who may be the subject of future epidemiological research using this tool. Therefore, this study aimed to assess: (i) the test–retest reproducibility of identification of data-driven DPs and hypothesis-driven DPs (diet quality scores), (ii) the consistency of data-driven DPs with diet quality scores in sex and age subgroups of Poles aged 15–65 years.

## 2. Materials and Methods

### 2.1. Ethical Approval

The study was approved by the Bioethics Committee of the Faculty of Medical Sciences, University of Warmia and Mazury in Olsztyn on 17 June 2010 (resolution no. 20/2010). Informed consent was obtained from adult participants and parents or legal guardians of under-aged participants (<18 years).

### 2.2. Participants and Study Design

A cross-sectional study was conducted in Poland in 2014–2015. Respondents were recruited non-randomly by several scientific centres in Poland. Participants came from rural and urban areas of the country and differed in socioeconomic status. Inclusion criteria were permission for participation in research, age in the range of 15–65 years, lack of diagnosed chronic diseases (self-declared by respondent). Initially, 527 respondents were recruited and completed the questionnaire twice. The database was verified and 23 participants (4.4% of the initial sample) were excluded due to: age of respondents under 15 or over 65 years (*n* 9), suffering from a chronic disease declared by a respondent (*n* 1), living in another area of the country (*n* 13). The final study sample included 504 healthy people aged 15–65 years (55.6% of females) from three cities (Olsztyn, Bydgoszcz, Poznan) and the surrounding towns and villages, therefore subjects from two macroregions of Poland (North and North-West) [35].

The data were collected using the self-administered version of the Dietary Habits and Nutrition Beliefs Questionnaire (KomPAN^®^). Two administrations of the questionnaire (test and retest) were completed with a two-week interval. Details of the main research design were presented in our previous paper [33].

### 2.3. Dietary Patterns

Dietary patterns were derived based on food consumption frequency data. The KomPAN^®^ questionnaire included 33 food items to assess habitual diet over the past year. The list of food items is presented in Table S2. These 33 food items covered all of the most important food groups in the Polish diet: grain products (4 items), fruit/vegetables/legumes/potatoes (5 items), dairy products (4 items), meat/fish/eggs (6 items), fats (3 items), beverages (7 items), sweets and other products (4 items). Each respondent reported habitual consumption of 33 food items by indicating one of the six frequency categories, which were converted to daily frequency expressed as times/day [36]: never (0.0), 1–3 times a month (0.06), once a week (0.14), a few times a week (0.5), once a day (1.0), a few times a day (2.0).

Dietary patterns were derived using two approaches: (i) hypothesis-driven—diet quality scores, (ii) data-driven—dietary patterns derived using the Principal Component Analysis (PCA).

#### 2.3.1. Diet Quality Scores

Two diet quality scores were developed: Pro-Healthy-Diet-Index-10 (pHDI-10) and Non-Healthy-Diet-Index-14 (nHDI-14) [36]. The scores were calculated by summation of the daily frequency of selected food group consumption (times/day): (i) pHDI-10 included 10 food items representing pro-healthy foods: wholemeal bread; buckwheat, oats, wholegrain pasta or other coarse-ground groats; milk; fermented milk beverages; fresh cheese curd products; white meat; fish; pulse-based foods; fruit; vegetables (the score range: 0–20 points), (ii) nHDI-14 included 14 food items representing less healthy foods: white bread; white rice, white pasta, fine-ground groats; fast foods; fried foods; butter; lard; cheese; cold meats, smoked sausages, hot-dogs; red meat; sweets; tinned meat; sweetened beverages; energy drinks; alcoholic beverages (the score range: 0–28 points), and unified to the range of 0–100 points for each of the diet quality scores. The diet quality scores were also analysed as categorical variables by dividing the scores into tertiles in respect to the total sample, sex and age groups. The test–retest reproducibility of the diet quality scores in healthy and unhealthy subjects as well as for two modes of administration of the questionnaire, i.e., interview-administered and self-administered, was described previously based on a total of 954 subjects [33]. In this study, the test–retest reproducibility of the diet quality scores was assessed in sex and age subgroups of healthy people who completed the self-administered version of the questionnaire (504 subjects).

#### 2.3.2. Data-Driven Dietary Patterns

Input variables for data-driven DPs were all 33 food items (times/day). The data were checked using the Kaiser–Meyer–Olkin (KMO) index to measure the sampling adequacy [37] and Bartlett’s test of sphericity [38]. The PCA performance is justified when the KMO is greater than 0.5 and Bartlett’s test is statistically significant (*p* < 0.05). Across the study groups, KMO values ranged from 0.571 to 0.741 in test data, and from 0.572 to 0.685 in retest data (Table S1). Bartlett’s test was significant in all study subgroups (*p* < 0.0001).

To derive DPs using the PCA, a varimax normalised rotation was used [39]. The Kaiser criterion (eigenvalues ≥ 1.00), the Cattel’s scree plot and the total variance explained were considered in selecting the best solution. Food items with factor loadings >|0.40| have been considered to be important in the interpretation and labelling of the DPs. Data-driven DPs were derived in the total sample and in each of the study subgroups (i.e., two sex groups and four age groups), from test data and retest data separately. Factor scores (DP scores), reflecting the subject’s adherence to the dietary pattern, were computed by summation of the products of the value of food item and the quotient of the corresponding factor loading and the eigenvalue of the factor [6].

### 2.4. Other Variables

Participants were grouped into sex categories (males, females) and four age categories (15–17, 18–24, 25–44 and 45–65 years). The age categories were chosen to distinguish people at different stages of life, nutritional needs and opportunities to meet these needs. Three sociodemographic variables were used to characterise the total study sample and each of the study subgroups: place of residence (3 categories), economic situation of the family (3 categories) and education level (adults only; 3 categories).

### 2.5. Statistical Analysis

The normality of variables was checked by the Kolmogorov–Smirnov test [40]. Mean values and standard deviations (SD) of food consumption frequency (times/day) and diet quality scores (points) by sex and age subgroups were calculated. A comparison analysis for continuous variables was performed using Mann–Whitney’s test (for two sex groups) or Kruskal-Wallis’ test (for four age groups) and for categorical variables using the chi^2^ test.

Test–retest reproducibility of the data-driven DPs and the diet quality scores in the total sample and sex and age subgroups were assessed using several analyses: (i) intraclass correlation coefficient (ICC), (ii) cross-classification analysis, (iii) the kappa statistic. The consistency of the data-driven DPs with the diet quality scores were evaluated using the following analyses: (i) Spearman’s correlation coefficient, (ii) cross-classification analysis, (iii) the kappa statistic. The percentage agreement and the kappa statistics (with a 95% confidence interval (CI)) were calculated for DPs categorised according to the tertile distribution of DP scores in each study group. Tucker’s congruence coefficient (CC) was used to assess the similarity of the data-driven DPs identified in both administrations of the questionnaire [41].

The ICC interpretation was as follows: poor (<0.5), moderate (0.5 to <0.75), good (0.75 to <0.9) and excellent (≥0.9) agreement [42]. The interpretation of Spearman’s correlation coefficient was as follows: weak (<0.3), moderate (0.3 to <0.5), good (0.5 to <0.7) and very good (≥0.7) strength of the correlation [40]. The interpretation of the agreement measured using kappa statistics: poor (0.0–0.20), fair (0.21–0.40), moderate (0.41–0.60), good (0.61–0.80) and very good (0.81–1.00) [43]. The kappa values above 0.40, as well as more than 50% of subjects classified into the same category and less than 10% grossly misclassified into opposite thirds, indicated an acceptable agreement [44]. Tucker’s congruence coefficient (CC) in the range 0.85–0.94 was interpreted as fair similarity and CC ≥ 0.95 was interpreted as good similarity (two components can be considered equal) [41].

Details on minimum sample size calculation were described previously [33]. The statistical analysis was performed using STATISTICA 13 (Dell Inc.; Tulsa, OK, USA; StatSoft Polska, Cracow, Poland) and PS IMAGO PRO 6.0 on an IBM SPSS Statistics 26 analytical engine (Predictive Solutions, Cracow, Poland). *p*-values < 0.05 were considered statistically significant.

## 3. Results

### 3.1. Participant Characteristics

The characteristics of the total sample and sex and age subgroups are presented in Table 1. Females comprised 55.6% of the total sample, and across age subgroups their share ranged from 41.5% to 68.5%. Considering age subgroups, 28.8% of participants were aged 15–17 years, 29.0% were aged 18–24 years, 21.2% were aged 25–44 years, and 21.0% were aged 45–65 years. Most of the participants declared having an average family economic situation (73.4% of the total sample) and higher education (53.1% of adults). Significant differences between sex groups were found in age (*p* = 0.0003), place of residence (*p* = 0.0034) and economic situation of the family (*p* = 0.0292). Significant differences between age subgroups were found in sex (*p* = 0.0003), place of residence (*p* < 0.0001) and education level (*p* = 0.0395).

Mean values of food consumption frequency (times/day) and diet quality scores (points) in the total sample and by sex and age subgroups in test data are presented in Table S2.

### 3.2. Data-Driven DP Characteristics

**Figure 1:**
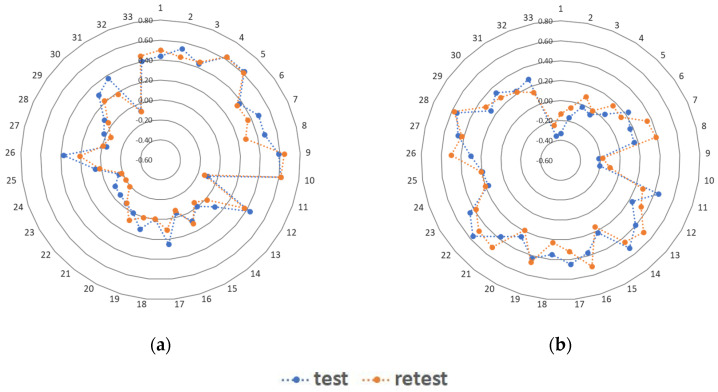
Diagrams of factor loadings of data-driven dietary patterns (DPs) identified in test data and retest data in the total sample: (**a**) ‘Prudent’ DP, (**b**) ‘Western’ DP. Food items: 1—Wholemeal bread; 2—Buckwheat, oats, whole grain pasta and other coarse-ground groats; 3—Milk; 4—Fermented milk beverages; 5—Fresh cheese curd products; 6—White meat; 7—Fish; 8—Pulse-based foods; 9—Fruit; 10—Vegetables; 11—White bread; 12—White rice, white pasta, fine-ground groats; 13—Fast foods; 14—Fried foods; 15—Butter; 16—Lard; 17—Cheese; 18—Cold meats, smoked sausages, hot-dogs; 19—Red meat; 20—Sweets; 21—Tinned meat; 22—Sweetened beverages; 23—Energy drinks; 24—Alcoholic beverages; 25—Vegetable oils, margarine, mixes of butter and margarine; 26—Eggs; 27—Potatoes (excluding chips and crisps); 28—Instant soups, ready-made soups; 29—Tinned vegetables; 30—Fruit juices; 31—Vegetable juices, fruit and vegetable juices; 32—Sweetened hot beverages; 33—Water.

Two major data-driven DPs were found separately from test data and retest data in the total sample and each of the sex and age subgroup: the ‘Prudent’ DP and the ‘Western’ DP. The ‘Prudent’ DP identified in the total sample from test data was characterised by the frequent consumption of 11 food items: fermented milk beverages (factor loading: 0.62), fresh cheese curd products (0.62), vegetables (0.62), fruit (0.59), buckwheat, oats, whole grain pasta, other coarse-ground groats (0.53), fish (0.48), pulse-based foods (0.47), wholemeal bread (0.44), milk (0.44), white rice, white pasta, fine-ground groats (0.44) and water (0.41) (Figure 1, Table S3). The ‘Western’ DP identified in the total sample from test data was characterised by the frequent consumption of eight food items: sweetened beverages (0.56), instant soups, ready-made soups (0.54), fried foods (0.52), potatoes (excluding chips and crisps) (0.46), energy drinks (0.45), cheese (0.45), white bread (0.44) and red meat (0.43) (Figure 1, Table S4).

The similarities and differences between the ‘Prudent’ DPs identified from test data and retest data in the sex and age subgroups are shown in Figure S1, and between the ‘Western’ DPs in Figure S2. The exact values of factor loadings of the ‘Prudent’ DPs identified in the total sample and all study subgroups in test data and retest data are presented in Table S3, and for the ‘Western’ DPs in Table S4. Eigenvalues and variance explained (%) are shown in Table S5. The total variance explained for both DPs identified in the total sample was 21.7% in the test, and 20.3% in the retest. Across the study subgroups, the total variance explained for both DPs derived from test data ranged from 21.1% in males to 24.7% in 15–17-year-olds, and from the retest data ranged from 20.1% in males to 24.1% in 15–17-year-olds.

### 3.3. Reproducibility of Data-Driven DPs

Tucker’s congruence coefficient (CC) indicated fair-to-good similarity of data-driven DPs between test and retest for all study subgroups, except for both DPs in males and ‘Western’ DP in the youngest age group (the CC < 0.85) (Table 2). For ‘Prudent’ DPs, the CC was 0.97 in the total sample, 0.65 in males and 0.92 in females; for age subgroups, it ranged from 0.87 in 15–17-year-olds to 0.96 in 18–24-year-olds. For ‘Western’ DPs, the CC was 0.94 in the total sample, 0.68 in males and 0.89 in females; for age subgroups, it ranged from 0.83 in 15–17-year-olds to 0.95 in 18–24-year-olds.

The ICC indicated moderate-to-good test–retest reproducibility for data-driven DPs, with the lowest values in males (Table 2). For ‘Prudent’ DPs, the ICC was 0.83 in the total sample, 0.56 in males and 0.81 in females; for age subgroups, it ranged from 0.75 in 45–65-year-olds to 0.86 in 18–24-year-olds. For ‘Western’ DPs, the ICC was 0.76 in the total sample, 0.57 in males and 0.67 in females; for age subgroups, it ranged from 0.61 in 15–17-year-olds to 0.82 in 25–44-year-olds.

The percentage agreement was significantly different between males and females for ‘Prudent’ DPs (test vs. retest), and by sex and age subgroups for ‘Western’ DPs (Table 3). For ‘Prudent’ DPs, the percentage of subjects classified into the same tertile in test data and retest data ranged from 53.1% in males to 74.2% in the total sample. For ‘Western’ DPs, the percentage agreement between test and retest ranged from 52.2% in males to 75.3% in 18–24-year-olds. The proportions of subjects grossly misclassified ranged from 0.0% to 7.1% in the study subgroups.

The kappa statistic showed fair-to-good agreement between test and retest for data-driven DPs, with the lowest values in males (Table 3). For ‘Prudent’ DPs, the kappa statistic ranged from 0.30 in males to 0.61 in the total sample. For ‘Western’ DPs, the kappa statistic ranged from 0.28 in males to 0.63 in 18–24-year-olds.

### 3.4. Reproducibility of Diet Quality Scores

The ICC indicated good test–retest reproducibility for diet quality scores (Table 2). Across the study subgroups, the ICC ranged from 0.84 in 45–65-year-olds to 0.88 in 25–44-year-olds for pHDI-10, and from 0.75 in females and 15–17-year-olds to 0.88 in 25–44-year-olds for nHDI-14.

The percentage agreement between test and retest for diet quality scores was not significantly different either by sex or by age subgroups (Table 3). The percentage of subjects classified into the same tertile in test and retest ranged from 74.3% in females to 79.4% in 25–44-year-olds for pHDI-10, and from 69.7% in 15–17-year-olds to 83.2% in 25–44-year-olds for nHDI-14. Proportions of subjects grossly misclassified ranged from 0.0% to 5.5% in the study subgroups.

The kappa statistic showed moderate-to-good agreement between test and retest for diet quality scores (Table 3). The kappa statistic ranged from 0.61 in females to 0.69 in 25–44-year-olds for pHDI-10, and from 0.54 in 15–17-year-olds to 0.75 in 25–44-year-olds for nHDI-14.

### 3.5. Consistency of Data-Driven DPs with Diet Quality Scores

Good-to-very good correlation was found between the data-driven DPs and diet quality scores in test data, with the lowest values in 18–24-year-olds (Table 2). The Spearman’s correlation coefficient between ‘Prudent’ DP and pHDI-10 ranged from 0.63 in 18–24-year-olds to 0.93 in the total sample and females, and between ‘Western’ DP and nHDI-14 it ranged from 0.60 in 15–17-year-olds to 0.81 in the total sample and males.

The percentage agreement between the data-driven DPs and diet quality scores was significantly different by age subgroups only (Table 3). For ‘Prudent’ DP and pHDI-10, the percentage of subjects classified into the same tertile ranged from 54.1% in 18–24-year-olds to 80.0% in females. For ‘Western’ DP and nHDI-14, the percentage agreement ranged from 53.4% in 18–24-year-olds to 72.9% in 25–44-year-olds. The proportions of subjects grossly misclassified ranged from 0.0% to 6.2% in the study subgroups.

The kappa statistic showed fair-to-good agreement between the data-driven DPs and diet quality scores (Table 3). For ‘Prudent’ DP and pHDI-10, the kappa statistic ranged from 0.31 in 18–24-year-olds to 0.70 in the total sample and in females. For ‘Western’ DP and nHDI-14, the kappa statistic ranged from 0.30 in 18–24-year-olds to 0.59 in 25–44-year-olds.

## 4. Discussion

The current findings showed that test–retest reproducibility was fair-to-good for data-driven DPs, and moderate-to-good for diet quality scores. The lowest reproducibility of data-driven DPs was found in males and in adolescents (for ‘Western’ DP). The reproducibility of diet quality scores tended to be higher in males than females (especially for nHDI-14) and was the highest in 25–44-year-olds. Considering the approach used to derive DPs, the reproducibility of diet quality scores was better than data-driven DPs. The consistency of data-driven DPs with diet quality scores was fair-to-very good, with the lowest values in young adults (18–24-year-olds). The consistency of pro-health DPs was higher in females than males, while for unhealthy DPs it tended to be higher in males. The reproducibility and consistency of pro-health DPs derived using two approaches (pHDI-10, ‘Prudent’ DP) tended to be better than unhealthy DPs (nHDI-14, ‘Western’ DP) in most study subgroups.

### 4.1. Reproducibility of Data-Driven DPs

Two major data-driven DPs were identified separately from test data and retest data in all study subgroups—‘Prudent’ DP and ‘Western’ DP. The test–retest reproducibility of data-driven DPs was fair-to-good. Intraclass correlations were moderate to good, and the kappa statistic indicated fair-to-good agreement for the DPs (in tertiles) between test and retest. There was an acceptable percentage agreement (≥50% of subjects classified into the same tertiles of DP) and proportions of grossly misclassified subjects in all study subgroups (<10%).

There are a limited number of studies assessing the test–retest reproducibility of data-driven DPs in other countries [6,28,29,30,31,32] and Poland [34]. Better reproducibility of pro-health than less healthy DPs was found in some of the studies [6,34]. Correlations for two major DPs derived from the test and retest of the 131-item FFQ conducted in American men were 0.70 for the ‘Prudent’ pattern and 0.67 for the ‘Western’ pattern [6]. In Polish females aged 13–21 years, test–retest reproducibility of DPs was assessed for two sets of data—non-aggregated (60 food items) and aggregated (25 food items) [34]. The reproducibility was better for pro-health DPs than less healthy DPs (traditional with a westernised profile), and non-aggregated than aggregated data [34]. In other studies, less healthy DPs were more reproducible than healthier patterns [28,30,31]. In Swedish women, test–retest correlations were lower for the ‘Healthy’ pattern than the ‘Western’ pattern or the ‘Drinker’ pattern [30]. In Iranian adults, the ICCs between test and retest were 0.72 for the ‘Iranian Traditional’ pattern and 0.80 for the ‘Western’ pattern [28]. In Japanese adults, the test–retest reproducibility of DPs was the lowest for the ‘Westernized Japanese’ pattern in men (r = 0.55) and the ‘Prudent Japanese’ pattern in women (r = 0.55), and the highest for the ‘Traditional Japanese’ pattern in men (r = 0.77) [32]. In Chinese adults, the reproducibility of the ‘Prudent’ pattern was lower than the ‘Processed food’ pattern [31]. In another study among Chinese adults, the reproducibility was the lowest for the ‘Nuts and sweets’ pattern and the highest for the ‘Animal and plant’ pattern [29].

In the literature, there is a distinction between the reproducibility of data-driven DPs identified at different time points (in the same sample) and in different studies (samples) within the same country or across countries [17,45,46]. DPs can be compared in the same sample between two time points in a relatively short period of time (≤1 year; test–retest reproducibility), in the same sample over longer time periods, on multiple occasions (stability over time) or between different studies at one time point (cross-study reproducibility) [17]. In the present study, fair-to-good similarity of data-driven DPs (CC ≥ 0.85) between two points in time (test vs. retest) was found for all study subgroups, except for males (both DPs) and the youngest age group (‘Western’ DP). It is difficult to compare the DP reproducibility (similarity) measured using the CC in the present study with other studies, which aimed to assess DP reproducibility using the CC, but between different samples within a country [45,46]. In comparing DPs derived in Spanish adult women from two different studies, the highest reproducibility was found for the ‘Western’ pattern (CC = 0.90), and lower for the ‘Prudent’ pattern (CC = 0.76) and the ‘Mediterranean’ pattern (CC = 0.77) [46]. In a systematic review of DPs derived in Japanese adults, it was shown that the reproducibility of pro-health DPs seems to be higher than less-healthy DPs within the country [45]. Based on high-quality data, the ‘Healthy’, ‘Prudent’ and ‘Japanese’ patterns were relatively reproducible within a country (CC: 0.80 to 0.89), while low reproducibility was found for the ‘Traditional Japanese’, ‘Western’ and ‘Traditional’ patterns (CC: 0.31 to 0.59) [45].

Data-driven DPs are not easily comparable between studies and should be interpreted with caution due to the methodological differences in DP identification [6,13,15,45]. It was found that the number of food items (or food groups) used in the analysis, type of analysis and rotation employed to extract DPs can influence the reproducibility of DPs [10,13,34]. It should be emphasised that data-driven DPs depend on the population, and their generalization and comparison with the results of studies conducted in other populations is limited [45]. A typical diet in Poland includes both animal-based and plant-based foods. Two distinct data-driven DPs were identified in the present study—‘Prudent’ DP and ‘Western’ DP. In other studies conducted in Poland, several DPs were derived, including the ‘Traditional’ pattern [2,47,48]. The ‘Traditional Polish’ DP was mainly characterised by the frequent consumption of white bread, potatoes, red meat, margarine/butter, and fried chicken [2]. The ‘Westernized Polish’ DP was one of the patterns identified in adolescents and was characterised by a higher consumption of mixed meat dishes, animal fats, sweets/desserts, vegetables, boiled potatoes and French fries/potato pancakes [49]. The westernisation of the diet has been observed in Poland and some other countries of the world [32,34,49,50]. As in the present study, the ‘Prudent’ (or ’Healthy’ DP) and ‘Western’ DP were derived in many other countries [5,6,12,30,32,34,46]. However, despite similar labels, the DPs derived in other populations may differ in the food composition of each type of pattern. For example, ‘Prudent Japanese’ DP was characterised by high intakes of vegetables, fruit and typical Japanese foods such as soy products, mushrooms, seaweed, oily fish and green tea, while the ‘Westernized Japanese’ DP was characterised by high intakes of bread, meat, processed meat, coffee, black tea, fruit juice, soft drinks, mayonnaise, and salad dressing [32]. Compared to Poland, the extent of diet westernisation seems to be less in Japanese adults [50]. The DPs derived in the present study were more similar to ‘Prudent’ and ‘Western’ patterns identified in the United States [6,7,12]. In the study of Hu et al. [6], the ‘Prudent’ DP was characterised by a high intake of vegetables, legumes, fruit, whole grains and fish/seafood, whereas the ‘Western’ DP was characterised by a high intake of processed meat, red meat, high-fat dairy products, eggs, butter and refined grains. Despite the differences in the composition of DPs across populations, the ‘Prudent’ or ‘Healthy’ DPs were generally related to lower body weight, disease incidence and mortality, while adverse effects were observed for ‘Western’ DPs [5,12,15].

### 4.2. Reproducibility of Diet Quality Scores

The test–retest reproducibility of diet quality scores was moderate-to-good. Test–retest reproducibility tended to be better for pHDI-10 than nHDI-14 across study subgroups, except for two age subgroups (18–24-year olds, 25–44-year-olds). A similar level of reproducibility of these diet quality scores (in tertiles) has been demonstrated previously in a study among Polish adolescents and adults aimed to assess the performance of interviewer- and self-administered versions of the KomPAN^®^ questionnaire in healthy people and a self-administered questionnaire in outpatients, showing better reproducibility of pHDI-10 than nHDI-14 [33]. The reproducibility of other pro-healthy (pHDI) and non-healthy (nHDI) diet indexes was evaluated in Polish schoolchildren aged 6–15 years [51]. The study showed better test–retest reproducibility of diet quality indexes in children with parents as proxy reporters than adolescents themselves, with a tendency toward higher results for nHDI than pHDI [51]. Test–retest reproducibility of various diet quality scores based on the dietary data collected using questionnaires has been evaluated in other countries [23,24,25,26,27]. Compared to the current results, relatively better reproducibility of diet quality scores was shown in Norwegian adolescents (percentage agreement: 87.6%) [24], although it was lower in New Zealand adolescents (60%) [27], Norwegian adults (69%) [23] and American men (r = 0.72) [25]. In Japanese adults, test–retest reproducibility was assessed for the Healthy Eating Index-2015 (HEI-2015) and Nutrient-Rich Food Index 9.3 (NRF9.3) estimated from two self-administered questionnaires—the Diet History Questionnaire (DHQ) and the Brief Diet History Questionnaire (BDHQ) [26]. Reasonable reproducibility was found for both questionnaires, with an ICC ranging from 0.53 to 0.77 [26]. Although the current findings seem to be similar to some of the above results, they cannot be entirely compared due to methodological differences, such as the type of questionnaire used to derive DPs, the composition of the diet quality score and the time interval between both administrations of the questionnaire [14,15,23].

The current study shows that diet quality scores were more reproducible than data-driven DPs. Since diet quality scores have a predefined list of components (food items), they are simpler to apply and compare across time, different populations and studies compared to data-driven DPs which are dependent on the population and subjective decisions of the researcher [12,45]. The input variables for data-driven DPs were all 33 food items from the KomPAN^®^ questionnaire covering the whole habitual diet, while two diet quality scores included 24 food items in total (i.e., 10 items for pHDI-10 and 14 items for nHDI-14) that were selected a priori based on the nutrition recommendations and literature [36]. The diet quality scores were designed to collectively capture the most important healthy/unhealthy features of a diet [36]. Lower test–retest reproducibility of the data-driven DPs could result from differences in the reporting of the frequency of consumption of some specific, characteristic for given DP food items that were not included in the diet quality scores. For example, significant differences in the mean values of daily frequency of food consumption between test and retest were found for ‘Vegetable juices, fruit and vegetable juices’ in most of the study subgroups (except for two age subgroups: 18–24 years, 45–65 years; data not shown).

### 4.3. Consistency of Data-Driven DPs with Diet Quality Scores

The consistency of data-driven DPs with diet quality scores was fair-to-very good. The consistency of pro-health DPs (‘Prudent’ DP and pHDI-10) tended to be higher than for unhealthy DPs (‘Western’ DP and nHDI-14) in the total sample and the study subgroups. Due to the methodological differences between the two approaches, their total compatibility was not expected. Hypothesis-driven DPs (diet quality scores) are designed based on prior knowledge, reflect adherence to dietary recommendations and have a constant, predefined list of food items [14]. Data-driven DPs showed the actual dietary habits as sets of different foods, including those that could not be captured using a hypothesis-driven approach [10,17]. However, data-driven DPs may not be as polarised as hypothesis-driven DPs, and sometimes they can combine opposite dietary characteristics [16]. In the present study, the ‘Prudent’ DPs identified separately in the study subgroups were mainly characterised by food items that were components of the pHDI-10 and only a few other food items (e.g., ‘Water’, ‘White meat’). Subjects with ‘Prudent’ DP consumed key healthy foods included in the pHDI-10 relatively often and avoided eating less healthy foods, which resulted in higher consistency of pro-health DPs. They seemed to follow the dietary recommendations strictly and consistently. Healthy eating habits may have resulted from greater nutrition knowledge [52,53] and health interest [54]. Lower consistency between unhealthy DPs derived using both approaches (‘Western’ DP and nHDI-14) indicates greater differences between the predefined composition of the nHDI-14 and unhealthy eating habits occurring in the studied subgroups. The ‘Western’ DPs derived in the present study were less explicit than ‘Prudent’ DPs and were characterised by various, mostly less healthy food items, some of which were components of the nHDI-14 and other food items which were not (e.g., ‘Potatoes’, ‘Instant soups, ready-made soups’).

### 4.4. Differences across Sex and Age Subgroups

This study shows that the test–retest reproducibility and consistency of the data-driven DPs and the diet quality scores in different subgroups of people varied. The percentage agreement of the subjects’ distribution by tertiles of ‘Prudent’ DPs (test vs. retest) differed significantly by sex, for ‘Western’ DPs by sex and age, while for diet quality scores no significant differences were noted. The percentage agreement between the data-driven DPs and diet quality scores differed significantly only by age. Considering all statistical analysis applied, the reproducibility of both data-driven DPs was better in females than males. Among age subgroups, the reproducibility was the lowest for ‘Western’ DP in adolescents. The reproducibility of diet quality scores tended to be better in males than females (especially for nHDI-14) and was the highest in 25–44-year-olds. The consistency of the data-driven DPs with diet quality scores was the lowest in young adults (18–24-year-olds). The consistency of pro-health DPs was better in females than males, but for unhealthy DPs it tended to be higher in males.

The better reproducibility of both data-driven DPs and consistency of pro-health DPs (‘Prudent’ DP with pHDI -10) noted in females can be explained by usually higher nutrition knowledge, health and weight concern and focusing on their food choices more than men [55,56,57]. In research conducted in 23 countries, it was shown that healthy eating was more important for women than for men [57]. According to a review of studies on dietary patterns derived empirically, it was demonstrated that more women than men have a healthier eating pattern [12]. For example, in a large sample of Greek adults, the Mediterranean pattern was associated with females, older age, higher education level and physical activity, while the Western pattern was associated with younger age, lower education level and current smoking [58]. A study conducted among Japanese adults showed that the test–retest reproducibility of the Nutrient-Rich Food Index 9.3 was better for females than males [26]. Higher reproducibility of diet quality scores in females than males was expected but not found in the present study. The study showed better reproducibility of nHDI-14 and the consistency of unhealthy DPs in males than females. Men could have less healthy but stable eating habits, which may facilitate the consistent reporting of food consumption. People with a stable diet and those more educated can reliably recall their dietary behaviours [19].

Lower reproducibility and consistency of DPs was found in younger age subgroups, especially for unhealthy DPs. Lower compliance between DPs could probably result from growing independence, peer influence, more out-of-home eating, and unstable eating habits as typical factors affecting dietary reporting in adolescents [59,60]. They are more prone to underreport their food consumption [59], which may be due to social desirability, body image concern and/or dieting [60]. The above reasons may explain difficulties in reliable reporting, particularly for less healthy food consumption, by the youngest respondents. The highest reproducibility of DPs was mostly found in 25–44-year-olds. Adults usually have more established eating habits than adolescents. However, in the oldest adults, the reproducibility of DPs may be lower due to impaired memory and difficulties in recalling their past dietary behaviours.

### 4.5. Implications for Future Studies

Assessment of the reproducibility and consistency of diet quality scores and data-driven DPs in different subgroups of people provides valuable information for researchers planning future epidemiological studies using this tool. In line with the literature, the current findings showed that the reliable assessment of dietary behaviours using a data-driven approach in males and adolescents can be more difficult than in females and adults. The diet quality scores were more reproducible than data-driven DPs and may be more suitable for dietary assessment, especially in men and adolescents. Since pro-health dietary patterns derived using two approaches were more consistent than unhealthy DPs, the assessment of healthy eating habits using this questionnaire seems to be more independent of the approach used. Ultimately, the choice of the approach should be made depending on the research purpose, i.e., whether the study is focused on identifying the real DPs occurring in a given population or assessing the habitual diet according to predefined criteria using diet quality scores.

Diet quality scores have a predefined list of components, which makes them easier to apply and compare between different populations and/or studies than data-driven DPs [12,45]. In the present study, the diet quality scores are based on a total of 24 out of 33 food items included in the KomPAN^®^ questionnaire [36], allowing the list of questions to be shortened. It can be beneficial for some future research due to shorter time, lower cost of research and less respondent burden when respondents are asked about 24 instead of 33 food items, gaining a chance to obtain higher response rate [61]. Furthermore, our findings indicate the possibility of modifying and improving the composition of diet quality scores. In addition to common food items in ‘Prudent’ DP and pHDI-10 (or ‘Western’ DP and nHDI-14), some other food items had a significant contribution (i.e., factor loadings > |0.40|) in data-driven DPs identified in most study subgroups. The current findings suggest that ‘Water’ should be considered an additional component of the pHDI-10, while ‘Instant soups, ready-made soups’ should be considered an additional component of the nHDI-14. Although data-driven DPs can capture some specific food items that were not included in the diet quality scores, it should be emphasized that data-driven DPs are population dependent, and their generalization and comparison with the results of studies conducted in other populations is limited [45].

### 4.6. Strengths and Limitations

The main strength of the present study is a comprehensive assessment of the test–retest reproducibility and consistency of dietary patterns across sex and age subgroups of Poles. Since the KomPAN^®^ questionnaire was developed for people in a wide age range (15–65 years), the data-driven DPs have been derived and analysed separately in four age subgroups. Therefore, factors potentially affecting the quality of dietary reporting that are typical for one subgroup (e.g., adolescents) did not affect the results and their interpretation in other age groups. The general tendencies, such as better reproducibility of diet quality scores than data-driven DPs or higher consistency of pro-health than unhealthy DPs, were observed in the total sample as well as in the study subgroups. Second, evaluation of the reproducibility and consistency of hypothesis-driven DPs (diet quality scores) and data-driven DPs allows future researchers to choose the optimal approach in nutritional assessment considering the sex and age of the subjects. Finally, various statistical tests were applied to gain comprehensive insights and strengthen the conclusions, according to the recommendations for reproducibility and/or validation studies [19,22,62].

However, there are several limitations of this study. Since the sample was not randomly recruited, there is a possibility of non-response bias [63]. In the voluntary sample, some groups of people, such as females, individuals with higher education levels and older individuals, can be overrepresented [63]. The present study was carried out in a large sample of adolescents and adults, from rural and urban areas in two macroregions of the country [35]. The sample included similar numbers of people in the subgroups of sex, age and place of residence. However, more than half of the participants had higher education level and they might be more health-conscious and have healthier dietary behaviours compared to the general population of Poles. Therefore, further research on the dietary behaviours of Poles aged 15–65 years in relation to sociodemographic factors in a representative national sample is needed. Second, although the questionnaire only includes 33 food items, it is comprehensive and a useful tool allowing us to distinguish healthy and unhealthy eating habits of Poles using two different approaches—hypothesis-driven and data-driven [4,64]. In respect to statistical analysis to identify data-driven DPs, the sample size of each of the study subgroups was relatively small, but sufficient for the PCA performance because the subject-to-item ratio for the study subgroups ranged from 3.2:1 to 8.5:1 [65,66]. Since only self-reported data can be collected with the questionnaire, social desirability bias and the misreporting of certain foods could potentially affect the results [22]. The consumption of some foods may be consistently misreported (e.g., less healthy food intake may be underestimated), leading to high reproducibility of certain food items, but not ensuring their validity. Thus, biomarker-based validity of the KomPAN^®^ questionnaire should be evaluated in future research. Finally, although the relatively short time interval (two weeks) between test and retest could contribute to a higher level of the reproducibility of DPs noted in the present study than in some other studies, a long time interval could reduce the reproducibility due to actual changes in the diet and underestimate the questionnaire performance [19,23]. A two-week interval was also used in other studies [10,23,27]. It is important to stress that the reproducibility assessment is limited—true changes in food consumption are impossible to distinguish from differences in the reporting of food consumption between test and retest (the questionnaire performance) [19,22].

## 5. Conclusions

The test–retest reproducibility of data-driven DPs and diet quality scores and their consistency were acceptable in most of the study subgroups, with a tendency to be higher for pro-health than unhealthy DPs. Data-driven DPs were more reproducible in females than males. Among age subgroups, the reproducibility was the lowest for the ‘Western’ DP in adolescents. The reproducibility of diet quality scores tended to be better in males than females (especially for nHDI-14) and was the highest in 25–44-year-olds. The current findings provide key information to choose the most appropriate approach to derive dietary patterns tailored to the study group and the purpose of future research. The KomPAN^®^ questionnaire can be recommended to use data-driven DPs and diet quality scores to describe habitual diets in people aged 15–65 years.

## Figures and Tables

**Table 1:** Sample characteristics (in the test) (%).

Variables	Total Sample		Sex			Age (Years)				
			Male	Female	*p*	15–17	18–24	25–44	45–65	*p*
	*n*	%	%	%		%	%	%	%	
Sample size (*n*)	504		224	280		145	146	107	106	
Sample percentage (%)		100.0	44.4	55.6		28.8	29.0	21.2	21.0	
Sex										0.0003
male	224	44.4	-	-		44.8	31.5	47.7	58.5	
female	280	55.6	-	-		55.2	68.5	52.3	41.5	
Age (years)					0.0003					
15–17	145	28.8	29.0	28.6		-	-	-	-	
18–24	146	29.0	20.5	35.7		-	-	-	-	
25–44	107	21.2	22.8	20.0		-	-	-	-	
45–65	106	21.0	27.7	15.7		-	-	-	-	
Place of residence					0.0034					<0.0001
village	183	36.3	43.3	30.7		34.5	38.4	44.9	27.4	
town	188	37.3	29.9	43.2		53.8	35.6	24.3	30.2	
city (>100,000 inhabitants)	133	26.4	26.8	26.1		11.7	26.0	30.8	42.5	
Economic situation of family					0.0292					0.1810
below average	30	6.0	8.5	3.9		8.3	2.1	6.5	7.5	
average	370	73.4	68.3	77.5		75.9	73.3	73.8	69.8	
above average	104	20.6	23.2	18.6		15.9	24.7	19.6	22.6	
Education level (adults) ^1^					0.2131					0.0395
primary/lower secondary	46	13.1	16.6	10.3		NA	13.7	8.4	17.0	
upper secondary	119	33.8	31.8	35.4			41.0	32.7	25.5	
higher	187	53.1	51.6	54.4			45.3	58.9	57.5	

^1^ the sample size varies due to the lack of data for this variable. *p*—significance level of chi^2^ test. NA—not applicable.

**Table 2:** Tucker’s congruence coefficient and correlation coefficients for dietary patterns derived from test data and retest data.

Variables	Total Sample	Sex		Age (Years)			
		Male	Female	15–17	18–24	25–44	45–65
Sample size (*n*)	504	224	280	145	146	107	106
Tucker’s congruence coefficient							
Prudent DP (test vs. retest)	0.97	0.65	0.92	0.87	0.96	0.94	0.91
Western DP (test vs. retest)	0.94	0.68	0.89	0.83	0.95	0.94	0.86
Intraclass correlation coefficient ^1^							
Prudent DP (test vs. retest)	0.83	0.56	0.81	0.79	0.86	0.84	0.75
Western DP (test vs. retest)	0.76	0.57	0.67	0.61	0.78	0.82	0.62
pHDI-10 (test vs. retest)	0.86	0.86	0.86	0.86	0.85	0.88	0.84
nHDI-14 (test vs. retest)	0.81	0.84	0.75	0.75	0.85	0.88	0.76
Spearman’s correlation coefficient ^2^							
Prudent DP vs. pHDI-10 (test)	0.93	0.89	0.93	0.91	0.63	0.88	0.90
Western DP vs. nHDI-14 (test)	0.81	0.81	0.78	0.60	0.66	0.79	0.75

*n*—sample size. DP—dietary pattern. pHDI-10—Pro-Healthy-Diet-Index-10. nHDI-14—Non-Healthy-Diet-Index-14. ^1^ all values of the intraclass correlation coefficient significant at *p* < 0.001. ^2^ all values of Spearman’s correlation coefficient significant at *p* < 0.05.

**Table 3:** Percentage agreement and kappa statistic (95% CI) according to the tertile classification of data-driven dietary patterns and diet quality scores.

Variables	Total Sample	Sex		*p*	Age (Years)				*p*
		Male	Female		15–17	18–24	25–44	45–65	
Sample size	504	224	280		145	146	107	106	
Prudent DP (test vs. retest)									
Total agreement	74.2	53.1	68.9	0.0002	67.6	66.4	72.0	65.1	0.5511
±1 category	23.4	39.7	29.3		29.0	31.5	28.0	31.1	
±2 category	2.4	7.1	1.8		3.4	2.1	0.0	3.8	
Kappa statistic	0.61	0.30	0.53		0.51	0.50	0.58	0.48	
(95% CI)	(0.56–0.67)	(0.20–0.39)	(0.45–0.62)		(0.40–0.63)	(0.38–0.61)	(0.45–0.71)	(0.34–0.61)	
Western DP (test vs. retest)									
Total agreement	70.8	52.2	63.2	0.0434	53.1	75.3	72.0	66.0	0.0020
±1 category	25.6	42.0	31.8		40.0	21.9	27.1	29.2	
±2 category	3.6	5.8	5.0		6.9	2.7	0.9	4.7	
Kappa statistic	0.56	0.28	0.45		0.30	0.63	0.58	0.49	
(95% CI)	(0.50–0.62)	(0.19–0.38)	(0.36–0.53)		(0.17–0.42)	(0.53–0.73)	(0.45–0.71)	(0.36–0.63)	
pHDI-10 (test vs. retest)									
Total agreement	78.2	78.1	74.3	0.4528	78.6	76.0	79.4	75.5	0.7638
±1 category	19.6	18.8	23.2		18.6	19.9	19.6	22.6	
±2 category	2.2	3.1	2.5		2.8	4.1	0.9	1.9	
Kappa statistic	0.67	0.67	0.61		0.68	0.64	0.69	0.63	
(95% CI)	(0.62–0.73)	(0.59–0.75)	(0.54–0.69)		(0.58–0.78)	(0.54–0.74)	(0.58–0.81)	(0.51–0.75)	
nHDI-14 (test vs. retest)									
Total agreement	77.4	77.2	71.4	0.2002	69.7	78.1	83.2	70.8	0.0907
±1 category	19.4	21.4	25.4		24.8	19.2	16.8	24.5	
±2 category	3.2	1.3	3.2		5.5	2.7	0.0	4.7	
Kappa statistic	0.66	0.66	0.57		0.54	0.67	0.75	0.56	
(95% CI)	(0.61–0.72)	(0.58–0.74)	(0.49–0.65)		(0.43–0.66)	(0.57–0.77)	(0.64–0.85)	(0.43–0.69)	
Prudent DP vs. pHDI-10 (test)									
Total agreement	79.8	78.6	80.0	0.5986	75.9	54.1	74.8	77.4	0.0001
±1 category	20.0	21.4	19.6		24.1	41.1	24.3	21.7	
±2 category	0.2	0.0	0.4		0.0	4.8	0.9	0.9	
Kappa statistic	0.70	0.68	0.70		0.64	0.31	0.62	0.66	
(95% CI)	(0.64–0.75)	(0.60–0.76)	(0.63–0.77)		(0.53–0.74)	(0.19–0.43)	(0.50–0.74)	(0.54–0.78)	
Western DP vs. nHDI-14 (test)									
Total agreement	65.5	66.5	62.9	0.6929	59.3	53.4	72.9	63.2	0.0432
±1 category	33.5	30.8	34.3		34.5	43.2	24.3	34.0	
±2 category	1.0	2.7	2.9		6.2	3.4	2.8	2.8	
Kappa statistic	0.48	0.50	0.44		0.39	0.30	0.59	0.45	
(95% CI)	(0.42–0.54)	(0.41–0.59)	(0.36–0.53)		(0.27–0.51)	(0.18–0.42)	(0.47–0.72)	(0.31–0.59)	

DP—dietary pattern. CI—confidence interval. pHDI-10—Pro-Healthy-Diet-Index-10. nHDI-14—Non-Healthy-Diet-Index-14. *p*—significance level of chi^2^ test.

## Supplementary Materials

The following are available online at https://www.mdpi.com/2072-6643/12/12/3598/s1, Figure S1: Diagrams of factor loadings of ‘Prudent’ dietary patterns identified in test data and retest data in the study subgroups: (**a**) males, (**b**) females, (**c**) 15–17-year-olds, (**d**) 18–24-year-olds, (**e**) 25–44-year-olds, (**f**) 45–65-year-olds, Figure S2: Diagrams of factor loadings of ‘Western’ dietary patterns identified in test data and retest data in the study subgroups: (**a**) males, (**b**) females, (**c**) 15–17-year-olds, (**d**) 18–24-year-olds, (**e**) 25–44-year-olds, (**f**) 45–65-year-olds, Table S1: Adequacy of data used to identify the data-driven dietary patterns (DPs) in the total sample and sex and age subgroups in test data and retest data, Table S2: Food consumption frequency (times/day) and diet quality scores (points) in the total sample and by sex and age subgroups in the test of the questionnaire (KomPAN^®^) (mean ± standard deviation), Table S3: Factor loadings of the Prudent dietary patterns identified in the total sample and sex and age subgroups in the test and the retest of the questionnaire (KomPAN^®^), Table S4: Factor loadings of the Western dietary patterns identified in the total sample and sex and age subgroups in the test and the retest of the questionnaire (KomPAN^®^), Table S5: Eigenvalues and variance explained (%) in the data-driven dietary patterns (DPs) in the total sample and sex and age subgroups in the test and the retest of the questionnaire (KomPAN^®^).
