# Supplementary material for: Data-Driven Dietary Patterns and Diet Quality Scores: Reproducibility and Consistency in Sex and Age Subgroups of Poles Aged 15–65 Years
Source: Nutrients. 2020 Nov 24;12(12):3598. doi: 10.3390/nu12123598 (PMC7759929; doi:10.3390/nu12123598)
Supplement: Supplementary file 1 [file nutrients-12-03598-s001.pdf]

Data-Driven Dietary Patterns and Diet Quality Scores: Reproducibility and Consistency in Sex and Age Subgroups of Poles Aged 15-65 Years

Joanna Kowalkowska, Lidia Wadolowska, Jolanta Czarnocinska, Grzegorz Galinski, Anna Dlugosz, Dorota Loboda and Magdalena Czlapka-Matyasik

Supplementary Materials

Table S1. Adequacy of data used to identify the data-driven dietary patterns (DPs) in the total sample and sex and age subgroups in test data and retest data.

Variables	Total sample	Sex		Age (years)			
		**Male**	**Female**	**15-17**	**18-24**	**25-44**	**45-65**
Sample size (*n*)	504	224	280	145	146	107	106
**Subject-to-item ratio**	15.3:1	6.8:1	8.5:1	4.4:1	4.4:1	3.2:1	3.2:1
**Test**							
KMO index [1]	0.741	0.688	0.680	0.636	0.657	0.571	0.603
Bartlett's test of sphericity	3133.2	1829.1	1962.0	1540.2	1471.8	848.8	995.6
p-value	<0.0001	<0.0001	<0.0001	<0.0001	<0.0001	<0.0001	<0.0001
**Retest**							
KMO index [1]	0.685	0.633	0.641	0.621	0.614	0.572	0.578
Bartlett's test of sphericity	3192.4	1921.4	1952.5	1453.3	1596.1	1012.5	961.2
p-value	<0.0001	<0.0001	<0.0001	<0.0001	<0.0001	<0.0001	<0.0001

[1] KMO – Kaiser-Mayer-Olkin index.

Table S2. Food consumption frequency (times/day) and diet quality scores (points) in the total sample and by sex and age subgroups in test data (mean ± standard deviation).

Food items	Total sample	Sex			Age (years)				
		Male	Female	p	15-17	18-24	25-44	45-65	p
Sample size (*n*)	504	224	280		145	146	107	106	
**pHDI-10** [1]	24.9±12.9	23.0±12.5	26.5±13.1	0.0007	25.5±14.6	27.2±13.1	22.6±11.0	23.5±11.7	0.0237
**Components of pHDI-10** [1]									
Wholemeal bread	0.56±0.62	0.55±0.64	0.57±0.61	0.3569	0.51±0.59	0.64±0.65	0.48±0.57	0.61±0.67	0.1630
Buckwheat, oats, whole grain pasta or other coarse-ground groats	0.25±0.31	0.22±0.32	0.27±0.31	0.0676	0.26±0.35	0.30±0.32	0.21±0.27	0.19±0.28	0.0085
Milk	0.68±0.64	0.60±0.61	0.75±0.66	0.0075	0.80±0.68	0.68±0.63	0.68±0.62	0.55±0.60	0.0031
Fermented milk beverages	0.50±0.48	0.43±0.46	0.55±0.49	0.0009	0.48±0.51	0.57±0.49	0.48±0.47	0.45±0.44	0.0555
Fresh cheese curd products	0.39±0.43	0.36±0.38	0.41±0.46	0.6419	0.41±0.50	0.39±0.41	0.37±0.42	0.36±0.34	0.8109
White meat	0.43±0.33	0.43±0.35	0.43±0.31	0.7809	0.46±0.44	0.44±0.31	0.43±0.27	0.40±0.22	0.6688
Fish	0.16±0.22	0.18±0.24	0.15±0.20	0.0715	0.16±0.24	0.19±0.28	0.13±0.11	0.17±0.17	0.1102
Pulse-based foods	0.13±0.20	0.14±0.24	0.12±0.17	0.8381	0.13±0.21	0.15±0.24	0.09±0.10	0.12±0.22	0.0374
Fruit	0.96±0.67	0.84±0.65	1.06±0.68	0.0001	0.98±0.74	1.05±0.71	0.81±0.56	0.99±0.62	0.0748
Vegetables	0.93±0.65	0.84±0.64	0.99±0.65	0.0025	0.92±0.70	1.03±0.69	0.84±0.59	0.88±0.55	0.1472
**nHDI-14** [2]	18.4±8.9	20.6±9.4	16.6±8.2	<0.0001	20.6±10.4	17.0±8.9	17.3±7.7	18.2±7.3	0.0095
**Components of nHDI-14** [2]									
White bread	0.90±0.74	0.97±0.74	0.85±0.73	0.0745	1.00±0.72	0.79±0.73	0.81±0.68	1.02±0.79	0.0113
White rice, white pasta, fine-ground groats	0.28±0.28	0.28±0.27	0.29±0.28	0.9802	0.31±0.30	0.35±0.32	0.22±0.20	0.23±0.21	0.0012
Fast foods	0.10±0.18	0.13±0.20	0.08±0.15	0.0062	0.16±0.28	0.11±0.14	0.08±0.08	0.04±0.06	<0.0001
Fried foods	0.35±0.29	0.39±0.30	0.32±0.28	0.0093	0.35±0.33	0.32±0.28	0.38±0.28	0.36±0.26	0.1776
Butter	0.71±0.76	0.77±0.78	0.67±0.75	0.1596	0.76±0.78	0.62±0.73	0.72±0.74	0.77±0.79	0.5194
Lard	0.05±0.15	0.07±0.19	0.03±0.10	0.0011	0.06±0.20	0.05±0.15	0.02±0.07	0.06±0.12	0.0005
Cheese	0.46±0.45	0.48±0.45	0.44±0.45	0.0495	0.54±0.58	0.46±0.44	0.43±0.33	0.38±0.32	0.5266
Cold meats, smoked sausages, hot-dogs	0.67±0.58	0.76±0.61	0.60±0.56	0.0005	0.71±0.66	0.51±0.50	0.65±0.50	0.84±0.60	<0.0001
Red meat	0.26±0.30	0.35±0.35	0.19±0.24	0.0000	0.21±0.35	0.23±0.32	0.28±0.24	0.35±0.24	<0.0001
Sweets	0.69±0.62	0.68±0.64	0.69±0.61	0.4665	0.81±0.67	0.66±0.66	0.66±0.59	0.58±0.51	0.0241

Food items	Total sample	Sex			Age (years)				
		Male	Female	p	15-17	18-24	25-44	45-65	p
Tinned meat	0.06±0.14	0.08±0.15	0.04±0.12	0.0000	0.09±0.18	0.05±0.16	0.05±0.08	0.04±0.06	0.1143
Sweetened beverages	0.33±0.51	0.44±0.57	0.23±0.43	0.0000	0.50±0.61	0.30±0.48	0.26±0.43	0.18±0.39	<0.0001
Energy drinks	0.12±0.33	0.17±0.38	0.08±0.27	0.0002	0.20±0.41	0.12±0.30	0.11±0.35	0.01±0.03	<0.0001
Alcoholic beverages	0.16±0.26	0.22±0.32	0.12±0.19	0.0002	0.07±0.20	0.19±0.26	0.19±0.26	0.22±0.29	<0.0001
**Other food items**									
Vegetable oils, margarine, mixes of butter and margarine	0.48±0.60	0.49±0.60	0.47±0.61	0.9070	0.34±0.52	0.37±0.52	0.63±0.67	0.66±0.68	<0.0001
Eggs	0.29±0.30	0.35±0.39	0.24±0.20	0.0021	0.27±0.39	0.30±0.32	0.28±0.21	0.29±0.22	0.0024
Potatoes (excluding chips and crisps)	0.44±0.31	0.48±0.34	0.40±0.28	0.0193	0.50±0.37	0.35±0.30	0.43±0.24	0.47±0.27	<0.0001
Instant soups, ready-made soups	0.07±0.15	0.09±0.18	0.06±0.12	0.1789	0.13±0.21	0.07±0.15	0.03±0.07	0.03±0.07	<0.0001
Tinned vegetables	0.16±0.26	0.18±0.29	0.14±0.22	0.2098	0.14±0.29	0.12±0.23	0.17±0.18	0.24±0.29	<0.0001
Fruit juices	0.48±0.55	0.46±0.53	0.50±0.57	0.8723	0.63±0.60	0.54±0.57	0.45±0.57	0.25±0.30	<0.0001
Vegetable juices, fruit and vegetable juices	0.16±0.32	0.16±0.30	0.17±0.33	0.8988	0.20±0.36	0.19±0.36	0.13±0.25	0.12±0.24	0.6922
Sweetened hot beverages	1.01±0.85	1.07±0.83	0.96±0.86	0.1121	1.05±0.75	0.89±0.85	1.24±0.86	0.89±0.91	0.0009
Water	1.28±0.81	1.23±0.80	1.32±0.81	0.2538	1.14±0.82	1.48±0.76	1.26±0.78	1.21±0.84	0.0022

[1] pHDI-10 – Pro-Healthy-Diet-Index-10 including 10 food items (the score range: 0-100). [2] nHDI-14 – Non-Healthy-Diet-Index-14 including 14 food items (the score range: 0-100).
p – significance level of Mann-Whitney's test (sex groups) or Kruskal-Wallis' test (age groups).

Table S3. Factor loadings of the Prudent dietary patterns identified in the total sample and sex and age subgroups in test data and retest data.

Food items [1]	Total sample		Sex				Age (years)							
			Male		Female		15-17		18-24		25-44		45-65	
	Test	Retest	Test	Retest	Test	Retest	Test	Retest	Test	Retest	Test	Retest	Test	Retest
**Fermented milk beverages**	**0.62**	**0.62**	**0.61**	**0.48**	**0.60**	**0.66**	**0.64**	**0.61**	**0.49**	**0.49**	**0.67**	**0.61**	**0.63**	**0.42**
**Fresh cheese curd products**	**0.62**	**0.60**	**0.67**	**0.42**	**0.56**	**0.62**	**0.66**	**0.61**	**0.56**	**0.56**	**0.62**	**0.47**	**0.51**	0.38
**Vegetables**	**0.62**	**0.62**	**0.53**	**0.56**	**0.65**	**0.61**	**0.60**	**0.66**	0.37	0.23	**0.59**	**0.62**	**0.53**	**0.66**
**Fruit**	**0.59**	**0.64**	**0.54**	**0.61**	**0.60**	**0.61**	**0.54**	**0.65**	0.33	0.30	**0.63**	**0.62**	**0.58**	**0.75**
**Buckwheat, oats, whole grain pasta, other coarse-ground groats**	**0.53**	**0.45**	**0.54**	**0.42**	**0.51**	**0.43**	**0.50**	0.17	0.13	0.13	**0.58**	**0.57**	**0.49**	0.35
**Fish**	**0.48**	0.36	**0.46**	**0.41**	**0.51**	0.25	**0.43**	0.19	**0.71**	**0.77**	0.09	0.04	**0.48**	**0.53**
**Pulse-based foods**	**0.47**	0.28	**0.52**	0.31	**0.45**	0.16	**0.48**	0.09	**0.66**	**0.71**	0.25	0.29	0.03	0.33
**Wholemeal bread**	**0.44**	**0.50**	0.33	**0.52**	**0.49**	**0.55**	0.37	**0.55**	0.26	0.35	**0.46**	0.37	**0.55**	**0.45**
**Milk**	**0.44**	**0.45**	**0.43**	0.15	**0.43**	**0.50**	**0.53**	**0.60**	0.21	0.29	**0.41**	0.27	**0.60**	0.34
**White rice, white pasta, fine-ground groats**	**0.44**	0.37	**0.56**	0.13	0.35	0.30	**0.50**	0.28	0.40	**0.42**	0.03	0.10	0.25	0.14
**Water**	**0.41**	**0.46**	0.27	0.31	**0.49**	**0.58**	**0.49**	**0.48**	0.00	-0.02	**0.43**	**0.47**	**0.45**	**0.47**
White meat	0.37	0.34	0.24	0.01	**0.48**	**0.41**	**0.61**	**0.55**	0.13	-0.02	0.07	0.22	0.38	0.23
Eggs	0.37	0.21	**0.47**	0.09	0.37	0.12	0.36	-0.02	**0.63**	**0.74**	0.12	0.04	0.25	0.31
Vegetable juices, fruit and vegetable juices	0.37	0.18	**0.46**	0.13	0.31	0.07	0.35	0.05	0.40	0.19	0.32	0.32	0.22	0.30
Fruit juices	0.30	0.22	**0.45**	0.13	0.19	0.07	0.22	0.25	**0.52**	0.33	0.22	0.22	0.09	0.16
Cheese	0.25	0.11	**0.44**	-0.07	0.10	0.04	**0.49**	0.24	0.39	0.37	-0.04	0.09	-0.08	-0.10
White bread	-0.10	-0.13	-0.10	**-0.45**	-0.06	-0.08	0.22	**0.42**	0.05	-0.08	-0.15	-0.26	-0.27	-0.35
Sweetened beverages	-0.07	-0.19	0.00	**-0.41**	-0.06	-0.23	0.01	0.07	0.31	0.26	-0.33	**-0.52**	-0.39	**-0.41**
Cold meats, smoked sausages, hot-dogs	0.00	-0.01	-0.01	-0.20	0.04	0.07	0.37	**0.51**	0.01	-0.12	-0.11	-0.32	-0.05	-0.17
Butter	0.09	0.12	-0.01	-0.11	0.17	0.19	0.30	**0.46**	0.11	0.02	0.14	0.01	0.01	-0.09
Sweets	0.00	0.08	0.17	-0.11	-0.13	0.10	0.13	**0.44**	-0.01	-0.09	0.03	-0.08	0.13	-0.09
Red meat	0.12	0.00	0.14	-0.22	0.20	0.04	0.25	0.06	**0.43**	0.32	-0.17	-0.01	-0.05	-0.16
Alcoholic beverages	-0.16	-0.19	-0.08	-0.29	-0.20	-0.19	-0.03	-0.07	0.01	-0.02	-0.37	**-0.58**	0.13	-0.06

Food items [1]	Total sample		Sex				Age (years)							
			Male		Female		15-17		18-24		25-44		45-65	
	Test	Retest	Test	Retest	Test	Retest	Test	Retest	Test	Retest	Test	Retest	Test	Retest
Tinned meat	-0.05	-0.05	-0.07	-0.17	0.01	-0.11	-0.09	-0.16	0.10	0.18	-0.20	**-0.48**	-0.17	-0.36
Fried foods	-0.01	-0.05	0.01	-0.27	0.02	-0.06	0.28	0.17	0.22	0.18	-0.27	-0.10	-0.23	**-0.42**
Tinned vegetables	0.09	0.04	0.08	0.05	0.10	-0.06	0.24	-0.02	0.05	0.07	-0.05	-0.05	0.26	0.33
Potatoes (excluding chips and crisps)	-0.04	-0.01	0.17	-0.10	-0.17	-0.16	0.05	0.08	0.30	0.39	0.03	-0.14	-0.10	0.03
Vegetable oils, margarine, mixes of butter and margarine	0.06	0.01	0.22	-0.02	-0.05	-0.08	0.15	0.07	0.27	0.37	0.33	0.26	0.05	0.02
Lard	-0.05	-0.07	-0.06	-0.21	0.02	-0.13	-0.03	-0.13	0.14	0.18	-0.28	-0.21	-0.04	-0.17
Energy drinks	-0.07	-0.20	0.06	-0.38	-0.12	-0.24	-0.06	-0.26	-0.01	0.16	-0.23	-0.36	-0.07	-0.21
Sweetened hot beverages	-0.08	-0.08	0.01	-0.26	-0.09	0.01	0.11	0.24	0.15	0.11	-0.14	-0.19	-0.39	-0.21
Fast foods	0.12	0.01	0.28	-0.13	0.04	-0.06	0.13	-0.01	0.39	0.33	-0.19	-0.21	-0.31	-0.29
Instant soups, ready-made soups	0.02	-0.05	0.05	-0.19	0.02	-0.20	0.03	-0.17	0.22	0.26	-0.27	-0.32	-0.12	-0.33

[1] sorted by factor loadings of DP identified in the total sample (test), then for next columns; factor loadings >|0.40| are marked in bold.

Table S4. Factor loadings of the Western dietary patterns identified in the total sample and sex and age subgroups in test data and retest data.

**Food items** [1]	**Total sample**		**Sex**				**Age (years)**							
			**Male**		**Female**		**15-17**		**18-24**		**25-44**		**45-65**	
	**Test**	**Retest**	**Test**	**Retest**	**Test**	**Retest**	**Test**	**Retest**	**Test**	**Retest**	**Test**	**Retest**	**Test**	**Retest**
**Sweetened beverages**	**0.56**	**0.48**	**0.58**	0.38	**0.49**	0.35	**0.57**	0.24	**0.55**	**0.57**	0.31	0.28	0.10	0.15
**Instant soups, ready-made soups**	**0.54**	**0.58**	**0.51**	**0.51**	**0.54**	**0.44**	**0.65**	**0.63**	**0.52**	**0.51**	0.08	-0.03	**0.46**	0.07
**Fried foods**	**0.52**	**0.45**	**0.46**	0.27	**0.63**	**0.58**	**0.45**	**0.43**	**0.50**	0.38	**0.45**	**0.57**	**0.62**	**0.52**
**Potatoes (excluding chips and crisps)**	**0.46**	**0.42**	**0.53**	**0.47**	0.32	**0.42**	**0.48**	0.37	0.28	0.17	**0.54**	**0.62**	**0.49**	**0.44**
**Energy drinks**	**0.45**	0.38	**0.47**	**0.42**	0.40	0.18	**0.66**	0.38	**0.47**	**0.44**	0.13	-0.09	0.16	0.28
**Cheese**	**0.45**	0.32	0.40	**0.43**	**0.44**	0.21	**0.41**	0.16	0.28	0.24	0.29	0.26	0.19	0.30
**White bread**	**0.44**	0.27	**0.50**	0.19	0.39	0.27	0.16	-0.02	**0.44**	0.40	**0.56**	**0.53**	**0.52**	**0.54**
**Red meat**	**0.43**	**0.46**	0.36	0.30	0.38	**0.56**	0.38	**0.50**	0.30	0.40	0.33	0.33	**0.50**	**0.46**
**Lard**	0.37	**0.51**	0.38	0.38	0.30	**0.51**	0.32	**0.57**	**0.44**	**0.49**	0.31	**0.41**	**0.51**	0.12
**Tinned meat**	0.38	**0.51**	0.38	0.36	0.29	**0.47**	**0.41**	**0.55**	0.29	**0.48**	0.25	0.19	**0.58**	0.34
**Fast foods**	0.39	**0.50**	0.36	**0.50**	0.32	**0.41**	0.34	**0.56**	0.32	0.38	**0.47**	0.26	-0.08	0.02
**Eggs**	0.30	**0.50**	0.20	**0.58**	0.16	0.38	0.26	**0.61**	0.10	0.14	0.18	0.09	0.20	0.18
Wholemeal bread	-0.33	-0.13	**-0.45**	-0.04	-0.28	0.00	-0.39	-0.17	**-0.49**	-0.36	-0.33	-0.08	-0.24	-0.29
White rice, white pasta, fine-ground groats	0.23	0.33	0.20	**0.62**	0.17	0.22	0.33	**0.49**	-0.17	-0.09	0.32	**0.43**	0.11	0.33
Fruit juices	0.34	0.27	0.32	**0.49**	0.39	0.28	0.23	0.10	0.08	0.02	**0.59**	**0.59**	0.08	0.11
Vegetable juices, fruit and vegetable juices	0.22	0.22	0.16	**0.47**	0.28	0.11	0.25	0.22	0.00	0.02	0.30	0.36	0.00	**0.42**
Fresh cheese curd products	0.04	0.16	0.01	**0.45**	0.01	0.16	-0.12	0.16	-0.22	-0.15	0.40	0.38	-0.16	**0.51**
Pulse-based foods	0.16	0.39	-0.08	**0.41**	0.33	**0.46**	0.31	0.37	-0.07	0.24	0.05	0.00	**-0.41**	**0.43**
Water	-0.35	-0.24	-0.30	-0.05	**-0.42**	-0.21	**-0.43**	-0.08	**-0.53**	**-0.51**	-0.17	-0.12	-0.08	-0.12
Fish	0.16	0.35	-0.04	0.31	0.25	**0.55**	0.07	**0.43**	0.12	0.07	-0.21	-0.09	-0.35	0.06
Vegetable oils, margarine, mixes of butter and margarine	0.20	0.21	0.10	0.16	0.28	0.28	**0.42**	0.20	0.06	0.08	0.15	0.39	0.37	0.03
Tinned vegetables	0.26	0.32	0.17	0.30	0.33	0.31	0.30	**0.43**	0.30	**0.49**	0.14	0.09	0.34	0.25
Fruit	-0.21	-0.18	-0.29	0.10	-0.12	-0.04	-0.24	-0.16	**-0.56**	**-0.54**	0.08	0.16	-0.25	-0.31

Food items [1]	Total sample		Sex				Age (years)							
			Male		Female		15-17		18-24		25-44		45-65	
	Test	Retest	Test	Retest	Test	Retest	Test	Retest	Test	Retest	Test	Retest	Test	Retest
Buckwheat, oats, whole grain pasta, other coarse-ground groats	-0.16	-0.06	-0.16	0.23	-0.18	-0.11	0.15	0.35	**-0.55**	**-0.48**	-0.23	-0.20	-0.30	-0.01
Alcoholic beverages	0.17	0.20	0.18	0.08	0.02	0.08	0.16	0.33	**0.52**	0.37	0.01	0.07	0.19	-0.02
Vegetables	-0.20	-0.10	-0.29	0.15	-0.14	-0.01	-0.20	-0.01	**-0.47**	-0.37	-0.27	-0.11	-0.27	-0.28
Cold meats, smoked sausages, hot-dogs	0.35	0.23	0.29	0.04	0.39	0.38	0.14	0.13	0.26	0.19	**0.49**	**0.49**	**0.60**	0.14
Butter	0.22	0.15	0.18	0.11	0.26	0.20	0.06	0.02	0.24	0.19	0.34	**0.44**	0.10	-0.29
Sweets	0.26	0.19	0.33	0.24	0.22	0.23	0.22	0.14	0.19	0.07	0.37	**0.41**	0.27	**0.48**
Sweetened hot beverages	0.28	0.13	0.34	0.06	0.21	0.10	0.09	-0.01	0.19	0.01	0.39	**0.41**	0.07	-0.05
White meat	0.23	0.14	0.27	0.22	0.19	0.24	0.05	0.20	0.11	0.06	0.21	-0.03	**0.43**	**0.52**
Milk	-0.02	0.09	0.12	0.38	-0.11	0.05	0.01	0.15	-0.32	-0.19	-0.22	-0.07	0.16	0.30
Fermented milk beverages	-0.06	-0.01	-0.17	0.23	0.04	0.11	-0.14	0.00	-0.39	-0.34	0.32	0.29	-0.03	0.12

[1] sorted by factor loadings of DP identified in the total sample (test), then for next columns; factor loadings >|0.40| are marked in bold.

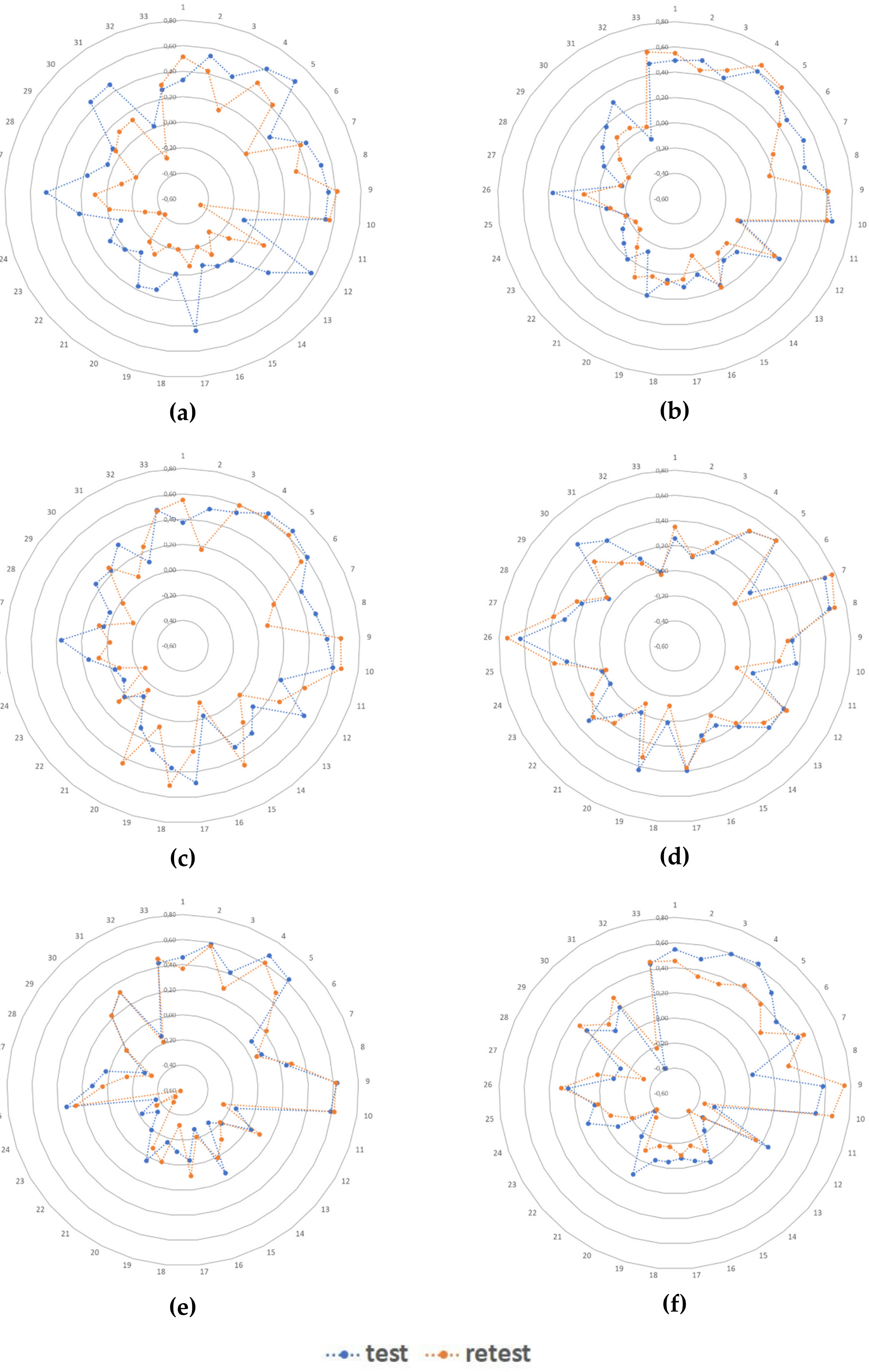
(a)
(b)
(c)
(d)
(e)
(f)
1 2 3 4 5 6 7 8 9 10 11 12 13 14 15 16 17 18 19 20 21 22 23 24 25 26 27 28 29 30 31 32 33
0,80
0,60
0,40
0,20
0,00
-0,20
-0,40
-0,60
test
retest

Figure S1. Diagrams of factor loadings of 'Prudent' dietary patterns identified in test data and retest data in the study subgroups: (**a**) males, (**b**) females, (**c**) 15-17-year-olds, (**d**) 18-24-year-olds, (**e**) 25-44-year-olds, (**f**) 45-65-year-olds. Food items: 1-Wholemeal bread; 2-Buckwheat, oats, whole grain pasta and other coarse-ground groats; 3-Milk; 4-Fermented milk beverages; 5-Fresh cheese curd products; 6-White meat; 7-Fish; 8-Pulse-based foods; 9-Fruit; 10-Vegetables; 11-White bread; 12-White rice, white pasta, fine-ground groats; 13-Fast foods; 14-Fried foods; 15-Butter; 16-Lard; 17-Cheese; 18-Cold meats, smoked sausages, hot-dogs; 19-Red meat; 20-Sweets; 21-Tinned meat; 22-Sweetened beverages; 23-Energy drinks; 24-Alcoholic beverages; 25-Vegetable oils, margarine, mixes of butter and margarine; 26-Eggs; 27-Potatoes (excluding chips and crisps); 28-Instant soups, ready-made soups; 29-Tinned vegetables; 30-Fruit juices; 31-Vegetable juices, fruit and vegetable juices; 32-Sweetened hot beverages; 33-Water.

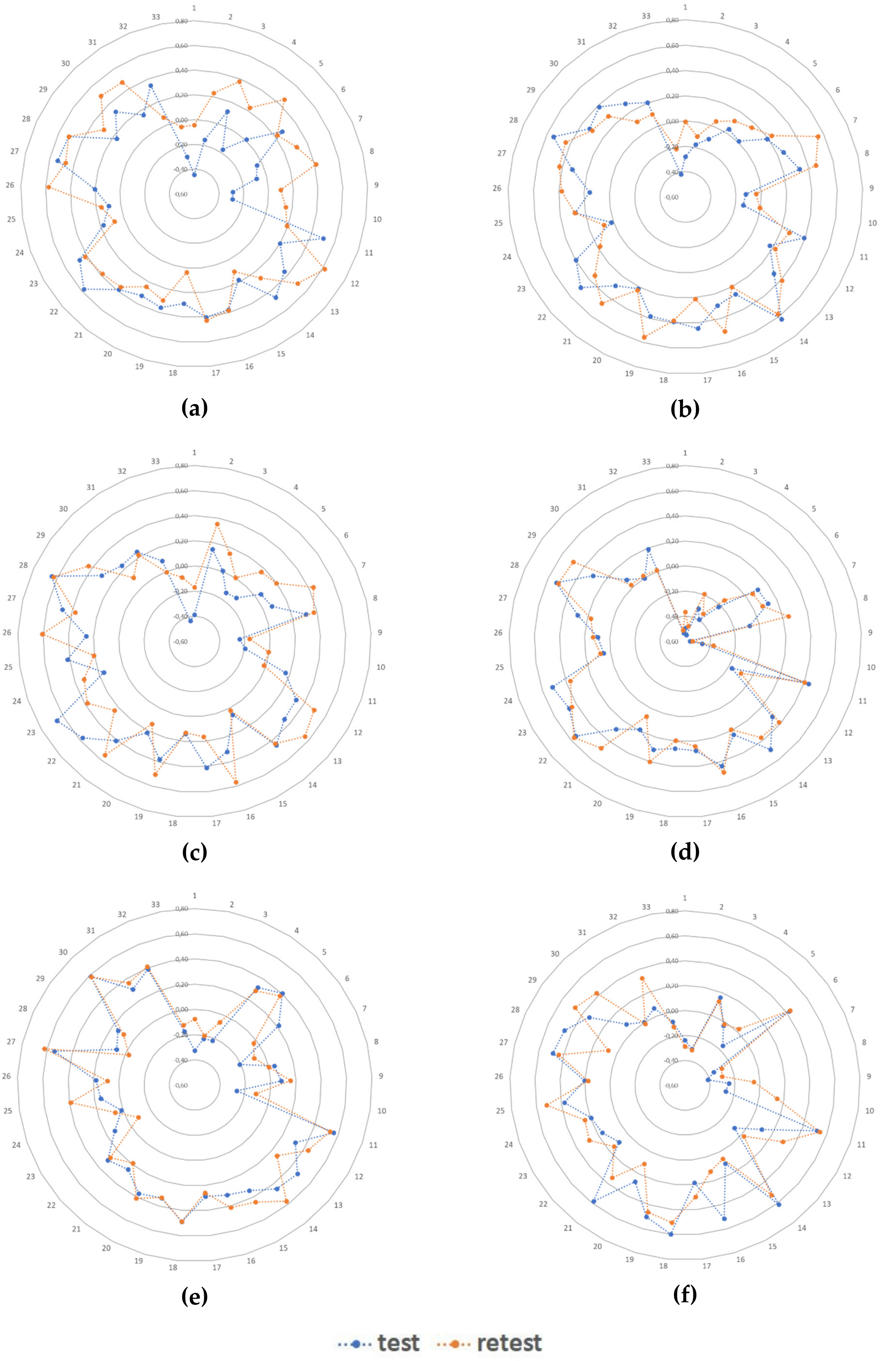
(a)
(b)
(c)
(d)
(e)
(f)
test
retest

Figure S2. Diagrams of factor loadings of 'Western' dietary patterns identified in test data and retest data in the study subgroups: (**a**) males, (**b**) females, (**c**) 15-17-year-olds, (**d**) 18-24-year-olds, (**e**) 25-44-year-olds, (**f**) 45-65-year-olds. Food items: 1-Wholemeal bread; 2-Buckwheat, oats, whole grain pasta and other coarse-ground groats; 3-Milk; 4-Fermented milk beverages; 5-Fresh cheese curd products; 6-White meat; 7-Fish; 8-Pulse-based foods; 9-Fruit; 10-Vegetables; 11-White bread; 12-White rice, white pasta, fine-ground groats; 13-Fast foods; 14-Fried foods; 15-Butter; 16-Lard; 17-Cheese; 18-Cold meats, smoked sausages, hot-dogs; 19-Red meat; 20-Sweets; 21-Tinned meat; 22-Sweetened beverages; 23-Energy drinks; 24-Alcoholic beverages; 25-Vegetable oils, margarine, mixes of butter and margarine; 26-Eggs; 27-Potatoes (excluding chips and crisps); 28-Instant soups, ready-made soups; 29-Tinned vegetables; 30-Fruit juices; 31-Vegetable juices, fruit and vegetable juices; 32-Sweetened hot beverages; 33-Water.

Table S5. Eigenvalues and variance explained (%) in the data-driven dietary patterns (DPs) in the total sample and sex and age subgroups in test data and retest data.

**Variables**	***n***	**Test**					**Retest**				
		**Eigenvalues**		**Variance explained (%)**		**Total variance explained (%)**	**Eigenvalues**		**Variance explained (%)**		**Total variance explained (%)**
		**Prudent DP**	**Western DP**	**Prudent DP**	**Western DP**		**Prudent DP**	**Western DP**	**Prudent DP**	**Western DP**	
Total sample	504	3.65	3.51	11.1	10.6	21.7	3.16	3.52	9.6	10.7	20.3
Sex											
male	224	4.08	3.41	12.4	10.3	22.7	2.98	3.89	9.0	11.8	20.8
female	280	3.68	3.28	11.2	9.9	21.1	3.29	3.34	10.0	10.1	20.1
Age (years)											
15–17	145	4.85	3.29	14.7	10.0	24.7	4.36	3.60	13.2	10.9	24.1
18–24	146	3.71	4.23	11.2	12.8	24.0	4.36	3.21	13.2	9.7	22.9
25–44	107	3.95	3.03	12.0	9.2	21.2	4.09	3.27	12.4	9.9	22.3
45–65	106	2.63	4.60	8.0	14.0	22.0	4.48	2.40	13.6	7.3	20.9

n – sample size.
